# Pharmacological neuroenhancement and the ability to recover from stress – a representative cross-sectional survey among the German population

**DOI:** 10.1186/s13011-018-0174-1

**Published:** 2018-10-22

**Authors:** Christiana Bagusat, Angela Kunzler, Jennifer Schlecht, Andreas G. Franke, Andrea Chmitorz, Klaus Lieb

**Affiliations:** 1 Department of Psychiatry and Psychotherapy, University Medical Center Mainz, Untere Zahlbacher Str. 8, 55131 Mainz, Germany; 2 German Resilience Center (DRZ) gGmbH, Untere Zahlbacher Str. 8, 55131 Mainz, Germany; 3 University of Applied Labour Studies, Bundesagentur für Arbeit, Seckenheimer Landstr. 16, D-68163 Mannheim, Germany

**Keywords:** Pharmacological neuroenhancement, Resilience, Stress coping, Illicit drugs, Prescription drugs

## Abstract

**Background:**

Pharmacological neuroenhancement (PNE) refers to the use of psychoactive substances without doctor’s prescription to enhance cognitive performance or to improve mood. Although some studies have reported that drugs for PNE are also being used to cope with stressful life situations, nothing is known about the relationship of PNE and resilience, i.e. the ability to recover from stress. This study aimed at investigating the relationship of PNE and resilience in the first representative population sample.

**Methods:**

A cross-sectional survey in a representative sample of 1128 adults (age ≥ 18 yrs.) living in Germany was conducted. The use of PNE and related attitudes, perceptions and behaviours were assessed by structured interviews and self-report questionnaires. Stepwise logistic regression with backward elimination was conducted to identify potential risk factors for PNE use.

**Results:**

Lifetime prevalence for the use of stimulating prescription drugs without medical indication was 4.3%, 10.2% for stimulating illicit drugs, 20.3% for mood modulating prescription drugs, and 23.4% for cannabis. Coping with stressful situations was more frequently reported as underlying motive for using stimulant or mood modulating prescription drugs than stimulating illicit drugs or cannabis. The individual perceived stress increased the risk of using stimulating prescription drugs (OR: 2.86; 95% Cl: 1.49–5.46) and the individual ability to recover from stress decreased the risk of using any substance for PNE and especially mood modulating prescription drugs (OR: .62; 95% Cl: .47–.81).

**Conclusions:**

The non-medical use of prescription drugs for PNE appears to be more prevalent in subjects who are less resilient to stress. Tailored resilience interventions that improve the ability to adapt to and recover from stressors may prevent the use of prescription medication for PNE. Further research should disentangle the association between psychological resilience and PNE as well as examine the efficacy of resilience interventions in the prevention of PNE.

**Electronic supplementary material:**

The online version of this article (10.1186/s13011-018-0174-1) contains supplementary material, which is available to authorized users.

## Background

Pharmacological neuroenhancement (PNE) refers to the use of psychoactive substances without medical indication to enhance cognitive performance or to improve mood [1–3]. Several studies have investigated the prevalence of PNE in different at risk populations such as pupils [4–6], students [4, 7–13], academics [14], chess players [15] or physicians [16]. International studies found divergent lifetime prevalences between 1 and 20% [4, 7, 16–20] which are dependent on the sample being investigated, the definition of PNE, the drugs questioned, and the survey technique used. Most studies have investigated PNE as a mode to enhance cognitive performance by the use of stimulating prescription or illicit drugs such as modafinil and methylphenidate or amphetamines, respectively. Other studies have also measured the use of mood modulating drugs such as antidepressants by non-depressed healthy subjects to improve mood or reduce nervousness and found lifetime prevalence rates between 5 and 15% [1, 16]. Data about the effectiveness of such substances are very different [2, 21].

Depending on the authors of scientific articles some consider PNE to be „a bad thing “which could harm individuals and change – in a negative way - society, however, others would favour the use of PNE drugs based on the ancient wish of mankind to enhance oneself [22, 23].

Research on the use of PNE as a strategy to cope with stressful life situations has been rather neglected so far. There is some evidence that PNE is associated with the level of perceived stress. Middendorff and colleagues [11] conducted a survey with over 7.000 students in Germany. Among those students who reported feeling no or a low pressure to perform at university, only 3% have already used prescription or illicit drugs for PNE at least once, whereas 9% of students feeling high pressure to perform have already used such medication or drugs [11]. In an online survey among the Swiss population, PNE was also positively associated with frequent stress in the past 12 months [10]. Other studies could demonstrate correlations between perceived stress and PNE among specific professions [24]. Surgeons’ pressure to perform at work and stress in private life were positively associated with PNE or mood enhancement [16]. In addition, work-related stress increased the willingness to use PNE-drugs in a study among university teachers [24]. Schröder and colleagues [25] compared users and non-users of PNE in physicians, publicists, advertising experts and programmers in Germany in the last 12 months. They found higher cognitive stress symptoms among PNE-users compared to non-users.

All these studies investigated the associations between perceived life stress and prevalence rates for PNE use. However, no study assessed the substance use for PNE in subjects who are able to recover from stress to varying degrees. This ability is closely related to the construct of psychological resilience, i.e., the well-observed phenomenon that many people do not or only temporarily become mentally ill despite significant adversity (e.g., [26–29]). Although previously considered as a stable personality trait (e.g., a “hardy” person), resilience is nowadays seen as modifiable outcome or dynamic process with personality as one of many risk or protective factors for maintaining or regaining mental health [30, 31]. Resilience is partially determined or predicted by multiple resilience factors [30], i.e., resources which protect a person from the potential negative effect of encountered stressors by modifying the individual’s response to stress and adversities [32, 33]. These include internal factors, such as (resilience-conducive) personality traits (e.g., optimism, hardiness), beliefs (e.g., self-efficacy), as well as external factors such as social support or socioeconomic status [34, 35]. Although there is evidence that some of the internal and external resilience factors are related to PNE use [11, 24, 36] the effect of the individual ability to recover from stress on PNE use is unknown so far.

In order to overcome limitations of previous surveys on PNE use regarding resilience factors, we performed a quantitative study on the relationship between the ability to recover from stress and PNE use in a representative sample of the German population. In addition, we assessed the association of PNE use with three well-evidenced resilience factors (self-efficacy, locus of control and optimism) [37, 38].

## Methods

### Participants and procedure

We conducted a cross-sectional representative survey of the German population between August 19th and September 19th 2016 regarding attitudes, perceptions and behaviours referring to PNE. Face-to-face interviews of approximately 30 min length were conducted in 1128 people at participant’s place (minimum age: 18 years). The individuals were selected by the “Institut für Demoskopie Allensbach” as they met criteria of the quota sample based on the German official statistics regarding central socio-demographic factors [39]. This procedure was chosen to increase the generalizability of the results for the German population. Participants were informed by professional interviewers about the objectives of the study, the procedure of data storage and confirmed their voluntary participation verbally. To help to assure confidentiality, there was no written consent. To ensure that each question is comprehensible, the standardized questionnaire was pretested and optimised.

The interviewers were trained uniformly to answer further questions if necessary for example if there were uncertainties in the understanding of terms such as “better cognitive performance”. Hence they especially clarified that the questions regarding PNE do not concern the intake of medication by doctor’s prescription. The questionnaire included a section assessing socio-demographic data such as gender, age, school education, current or last professional position, employment status, working hours, shift work, federal state of residence, size place of residence and soft enhancer intake.

Also there were questions regarding the perception of the topic PNE in the media, ethical questions and socio-psychological aspects which will be published separately.

Beside questions that were directly asked by the interviewer, the questionnaire also included a section that had to be completed written and returned hidden in an envelope to the interviewer to increase the reliability of the prevalence rates of PNE use. As these questions referred to the individual consumption of psychoactive substances to increase individual performance, this procedure guaranteed confidentiality for the respondent regarding these sensitive questions. The study was approved by the local Ethics Committee (Landesärztekammer Rheinland-Pfalz, No 837.209.14, 9448F), and there was no remuneration of the participating subjects.

### Assessment of prevalence of PNE and reasons and goals for intake

The questionnaire to assess the prevalence of PNE as well as goals and attitudes towards PNE by our group was based on experiences with earlier surveys [4, 16] and other published data.

#### Assessment of prevalence of PNE

To estimate the prevalence of PNE, we asked two main questions: The first question referred to enhancement by the intake of *freely available* substances that can be bought in supermarkets or pharmacies without any prescription such as energy drinks, *Ginkgo biloba* or caffeine tablets. The interviewer asked: *“There are various substances mentioned on this list. Are there any that you have taken or are currently taking to improve your mental performance, improve your mood, relieve anxiety or nervousness, or manage stress? You only have to tell me the corresponding numbers from the list.”* The second question referred to the use of prescription and/or illicit drugs. It was assessed within the self-completed anonymous paper-and pencil-section. Out of 1128 respondents, 86 had never heard of the phenomenon of PNE. Thus they were assigned to the group of “non-users” in the further analysis. Hence 1042 had been asked for their use of prescription/illicit substances for PNE.

Here, the interviewer asked: *“Here are some drugs that can be used to improve cognitive performance, improve mood, relieve anxiety or nervousness, or to manage stress. Please tick every drug if you have already taken it for the above-mentioned purpose without medical indication. If you have already taken a drug, please indicate when and how often.”* The list of substances included prescription drugs, such as methylphenidate (e.g., Ritalin®) or modafinil, and illicit substances such as cocaine or amphetamines. All substances were clustered into three substance groups (stimulants, mood enhancer and cannabis). For a more differentiated consideration “stimulants” were divided into “prescription stimulants” and “illicit stimulants” for further analysis. If one substance of a substance group was used at least once for PNE, the respondent was asked to answer further questions about the use. If more than one substance of a substance group was used, it was asked to choose the most important substance of that group and the respondent had to answer further questions about the use only for that substance of the group.

#### Assessment of reasons and goals for PNE

Beside the frequency of intake, participants were also asked for the reason of their use and which goals they pursued by the intake of the substance. The two questions were asked for one substance of each substance group. If they used multiple substances of one group (stimulants, mood enhancer, cannabis) they were asked to indicate reasons and goals only for the use of the most important one. They were asked: *“For what reason did you take this drug? Please tick for every reason stated to what extend this applies to you.”* and *“What were you trying to achieve by taking this drug? Please tick for every reason stated to what extend this applies to you.”* Users could rate the importance of different reasons and goals for the intake of the substance on a five-point Likert scale (1 = I totally agree; 5 = I totally disagree) e.g.*, “I wanted to improve mood” or*” *I wanted to be able to handle stressful situations better*”. The coding of items was reversed (1 = *I totally disagree*; 5 = *I totally agree*) before calculating mean values thus higher values mean higher importance of the reason or goal for the substance use. As individuals might be part of different substance groups there are no *p*-values to estimate statistical significance of mean values.

### Questionnaires to assess the ability to recover from stress, perceived stress and related psychological resilience factors

To allow for analyses of the association between PNE and the ability to cope with stress, different assessment scales were used:

#### Brief Resilience Scale (BRS) [40]

The scale consists of six items assessing self-ratings of the individual ability to recover from stress despite significant adversity (item 1” I tend to bounce back quickly after hard times”, item 2” I have a hard time making it through stressful events”, item 3” It does not take me long to recover from a stressful event”, item 4” It is hard for me to snap back when something bad happens”, item 5” I usually come through difficult times with little trouble” and item 6” I tend to take a long time to get over set-backs in my life”). The items are rated on a five-point Likert scale (1 = *strongly disagree;* 5 = *strongly agree).* Item 1, 3, and 5 are positively phrased; items 2, 4, and 6 are negatively phrased. The coding of the negatively phrased items is reversed in order to calculate the mean (range: 1–5) of the six items [40]. Higher values indicate a higher ability to recover from stress. We used the German version of the instrument, which was recently validated in a population of *n* = 2.609 German participants [41]. The psychometric data of the BRS ratings of the sample investigated here were part of this validation study.

#### Perceived Stress Scale (PSS-4) [42]

The PSS-4 consists of four items measuring the individual evaluation of stressful situations in the previous 12 months (item 1 “How often have you felt that you were unable to control the important things in your life?”, item 2.

“How often have you felt confident about your ability to handle your personal problems?”, item 3 “How often have you felt that things were going your way?”, item 4 “How often have you felt difficulties were piling up so high that you could not overcome them?”). The items are rated on a five-point Likert scale (1 = *never*; 5 = *very often*). Item 2 and 3 are reverse coded and were recoded for the analysis. For each subject, sum scores across all items are calculated (range: 0–16). Higher values indicate more perceived stress. We used the German version of the scale [43].

#### Short Scale for Measuring General Self-efficacy Beliefs (ASKU) [44]

The questionnaire consists of three positively worded items assessing self-rated confidence in the individual ability to achieve intended results *(“I can rely on my own abilities in difficult situations”, “I am able to solve most problems on my own”, “I can usually solve even challenging and complex tasks well”*) rated on a five-point Likert scale (1 = *does not apply at all*; 5 = *applies completely*). Mean scores are used for analysis (range: 1–5). Higher values indicate higher self-efficacy.

#### Short Scale for the Assessment of Locus of Control (IE) [45]

The four-item scale assesses internal and external control beliefs (internal control beliefs: *“I’m my own boss”, “If I work hard, I will succeed”*; external control beliefs: *“Whether at work or in my private life: What I do is mainly determined by others”, “Fate often gets in the way of my plans”*). The questions are rated on a five-point Likert scale (1 = *does not apply at all*; 5 = *applies completely*). Mean scores for internal or external control are calculated (range: 1–5). Higher values indicate higher internal (items 1 and 2) or external (items 3 and 4) control beliefs.

#### Optimism-Pessimism-2 Scale (SOP-2) [46]

The questionnaire consists of two items assessing self-rated optimism *(“How optimistic are you in general?”*) and pessimism *(“How pessimistic are you in general?”*). The questionnaire uses a seven-point Likert scale (optimism: 1 = *not at all optimistic*; 7 = *very optimistic;* pessimism: *1 = not at all pessimistic; 7 = very pessimistic*). To calculate the mean of the two items, reverse scoring of the item pessimism is used (range: 1–7).

### Statistical analyses

Analyses were performed with SPSS for Windows, Version 17.0. To guarantee representativeness to the highest possible standard, data of respondents included in the final sample was weighted for the area and federal states of Germany, size of the town, gender, school education, age and profession. N values of weighted data were rounded. Differences between users or subgroups of users and non-users in sociodemographic variables (gender, age, education, current or last professional position, employment status, working hours, shift work, federal state of residence, size place of residence and soft enhancer intake) were analysed using chi^2^-test, Fisher’s exact test, t-test and Welch test. Variables referring to the importance of reasons and goals were recoded for analysis (1 = *I totally disagree*; 5 = *I totally agree*). Means are reported with standard deviations (SD).

In order to assess the associations between PNE use and the ability to recover from stress, perceived stress and resilience factors (self-efficacy, control beliefs and optimism), stepwise logistic regression with backward elimination was conducted to determine predictors of PNE consumption using the most parsimonious model. Prior to multivariate analyses, means of users (and respective subgroups) with nonusers were compared using t-tests for continuous variables to assess the associations between each of the potential predictors (BRS, PSS-4, ASKU, IE internal, IE external, SOP-2) with PNE use. To test for multicollinearity, associations between the predictor variables (Pearson correlations) were examined and the variance inflation index (VIF) calculated. According to the literature, the correlations should not exceed .80, the VIF should not exceed 10 [47].

In the multivariate model, all predictors (BRS, PSS-4, ASKU, IE internal, IE external, SOP-2) were excluded stepwise. In order to control for potential confounders, sociodemographic variables (gender, age, education, current or last professional position, employment status, working hours, shift work, federal state of residence, size place of residence, soft enhancer intake) with a statistically significant mean difference between users or subgroups of users and non-users (*p* < .1) were included at once (method: enter) from the first step on in the model. Continuous variables (age, BRS, PSS-4, IE external, IE internal, SOP-2 and ASKU) were z-transformed. The significance level was *p* < 0.05.

## Results

### Sample characteristics

The total sample consists of *n* = 1128 subjects. Table 1 gives sociodemographic information for users and non-users of PNE.

**Table 1:** Sociodemographic characteristics for users and non-users in the representative survey of the German population

	Any medication or drug (*n* = 435)				Non-User (*n* = 686)				*p*-value
	Weighted^a^			Unweighted	Weighted^a^			Unweighted	
	% (n)	95% CI		% (n)	% (n)	95% CI		% (n)	
		min	max			min	max		
Age	47.7 (17.7)	46.0	49.3	48.0 (17.6)	53.4 (18.1)	52.0	54.8	52.9 (17.9)	0.01
Gender									0.06
Male	51.7 (225)	47.0	56.4	49.9 (217)	46.0 (316)	42.3	49.7	45.9 (315)	
Female	48.3 (210)	43.6	53.0	50.1 (218)	54.0 (371)	50.3	57.7	54.1 (372)	
Education									0.82
No formal degree	1.4 (6)	0.3	2.5	1.2 (5)	1.6 (11)	0.7	2.6	1.3 (9)	
Secondary modern school^b^	31.3 (133)	26.9	35.7	24.4 (104)	33.2 (227)	29.7	36.8	26.1 (178)	
Middle school^c^	30.4 (129)	25.9	34.7	31.5 (134)	30.9 (211)	27.4	34.4	31.2 (213)	
University-entrance diploma^d^	17.7 (75)	14.0	21.3	23.7 (101)	17.7 (121)	14.9	20.6	22.9 (156)	
University degree	19.3 (82)	15.5	23.1	19.2 (82)	16.5 (113)	13.8	19.3	18.5 (126)	
Current or last professional position									0.23
No employment yet	5.3 (23)	3.2	7.4	5.8 (25)	4.5 (31)	3.0	6.1	5.0 (34)	
Skilled worker	14.4 (62)	11.1	17.7	14.4 (62)	14.2 (97)	11.6	16.8	13.5 (92)	
Executive employee	10.7 (46)	7.7	13.6	10.9 (47)	14.2 (97)	11.6	16.8	14.5 (99)	
Non-executive employee	43.3 (187)	38.6	48.0	42.1 (182)	44.0 (300)	40.3	47.7	43.4 (296)	
Civil servants	5.6 (24)	3.4	7.7	6.7 (29)	6.5 (44)	4.6	8.3	7.6 (52)	
Self-employed	6.0 (26)	3.8	8.3	6.5 (28)	3.4 (23)	2.0	4.7	3.7 (25)	
Other	14.8 (64)	11.5	18.2	13.7 (59)	13.2 (90)	10.7	15.7	12.3 (84)	
Shift work									0.14
Yes	15.4 (44)	11.2	19.6	15.5 (44)	20.0 (75)	15.9	24.0	19.7 (74)	
No	84.6 (241)	80.4	88.8	84.5 (240)	80.0 (301)	76.0	84.1	80.3 (302)	
Weekly working hours									0.73
Currently not working	34.0 (147)	29.6	38.5	34.0 (147)	44.8 (307)	41.1	48.5	44.8 (307)	
< 20 h	6.7 (19)	3.8	9.6	6.3 (18)	4.3 (16)	2.2	6.4	4.3 (16)	
20–29	8.8 (25)	5.45	12.1	8.5 (24)	9.9 (37)	6.9	13.0	9.4 (35)	
30–40	50.5 (144)	44.2	56.3	50.4 (143)	50.4 (188)	45.3	55.5	49.9 (186)	
41–50	24.9 (71)	19.9	29.9	25.4 (72)	25.7 (96)	21.3	30.2	26.5 (99)	
> 50	9.1 (26)	5.8	12.5	9.5 (27)	9.7 (36)	6.7	12.7	9.9 (37)	
Size place of residence (inhabitants)									0.01
< 2.000	7.8 (34)	5.3	10.3	6.9 (30)	8.3 (57)	6.2	10.4	6.6 (45)	
2.000–20.000	29.7 (129)	25.4	34.0	29.2 (127)	37.3 (256)	33.7	40.9	34.5 (237)	
20.000–100.000	25.8 (112)	21.6	29.9	26.9 (117)	28.7 (197)	25.3	32.1	31.7 (218)	
> 100.000	36.8 (160)	32.3	41.3	37.0 (161)	36.8 (176)	32.3	41.3	27.2 (187)	
Soft enhancer intake									0.01
yes	86.3 (372)	83.1	89.6	86.3 (372)	49.5 (334)	45.7	53.3	50.4 (341)	
no	13.7 (59)	6.6	20.8	13.7 (59)	50.5 (341)	46.7	54.3	49.6 (336)	

*N* = 1128. Data are given in % with numbers of subjects in parentheses. For age, means with SD in parentheses are given. ^a^weighted according to the distribution of the general population in Germany as reported by the German Office of National Statistics; ^b^equivalent to German “Hauptschule” degree after 9 years of formal education; ^c^equivalent to German “Realschule” degree after 10 years of formal education; ^d^equivalent to German general or subject-specific. “Hochschulreife” or “Fachhochschulreife” degree (entrance qualifications for university or university of applied sciences) after 11. Twelve or 13 years of formal education; *p*-values: statistically significant differences between the respective group of users and non-users (α = .05); weighted n is rounded; weighted % refers to valid answers

### Prevalence rates for the use of substances for PNE

Most participants were familiar with the phenomenon of taking drugs or other substances for PNE (*n* = 1042; 92.4%). Table 2 summarizes the prevalence rates of the use of prescription and illicit drugs for PNE in the representative sample.

**Table 2:** Use of prescription and illicit drugs to enhance cognitive performance or mood without medical indication

Substance group / Single substances^a^	n^b^	Lifetime %^c^ (n)	Last year %^c^ (n)	Last month %^c^ (n)	Last week %^c^ (n)
Stimulating prescription drugs	1115	**4.3 (48)**	**2.2 (25)**	**0.8 (9)**	**0.3 (4)**
Prescription drug containing amphetamines	1112	1.7 (19)	0.8 (9)	0.3 (4)	–
Methylphenidate	1110	2.2 (25)	1.1 (12)	0.3 (3)	0.1 (1)
Anti-dementia drug	1110	1.0 (11)	0.6 (6)	0.2 (2)	0.2 (2)
Modafinil	1.107	0.4 (4)	0.1 (1)	0.1 (1)	0.1 (1)
Stimulating illicit drugs	1118	**10.2 (114)**	**3.8 (42)**	**1.3 (14)**	**0.6 (6)**
Cocaine	1114	6.1 (68)	1.9 (21)	0.3 (4)	0.1 (1)
Amphetamines	1115	6.9 (77)	2.5 (28)	0.8 (9)	0.3 (4)
Meth-Amphetamines	1111	2.0 (22)	0.6 (6)	0.1 (1)	0.1 (1)
Mood modulating prescription drugs	1110	**20.3 (225**)	**10.6 (118)**	**5.9 (66)**	**5.6 (62)**
Anti-depressant	1096	8.5 (93)	4.0 (44)	1.6 (18)	1.5 (16)
Beta blocker	1080	8.5 (92)	5.2 (56)	4.1 (45)	4.0 (43)
Benzodiazepines	1088	8.9 (98)	3.5 (38)	0.9 (10)	0.7 (8)
Cannabis	1109	**23.4 (260)**	**8.6 (94)**	**3.7 (40)**	**2.8 (30)**
Any medication or drug	1121	38.8 (435)	19.1 (214)	10.1 (113)	8.5 (95)

*N* = 1128

^a^As multiple selections were possible and some individuals used several substances, values could not be added up per substance group; ^b^N refers to valid values, i.e. all observations without missing values in the respective question; ^c^Weighted according to the distribution of the general population in Germany as reported by the German office of national statistics, n refers to the absolute frequency and % refers to the relative frequency of participants that have taken the respective substance ever in their life, in the last year, last month or last week

As one person is assigned to a substance-group via lifetime use of relevant substances, it is possible that individuals are part of several clustered substance-groups (Table 2).

Overall, lifetime use (“at least once”) of any prescription or illicit drug for cognitive or mood enhancement was 38.8%, with a last year, last month, and last week prevalence of 19.1%, 10.1%, and 8.5%, respectively (Table 2). Regarding different substance categories, the highest lifetime prevalence was found for cannabis (23.4%) followed by mood modulating prescription drugs (20.3%) and stimulating illicit drugs (10.2%). Stimulating prescription drugs had the lowest lifetime prevalence (4.3%) compared to the other substance groups (see Table 1). However, last year, last month and last week prevalence was higher for mood modulating prescription drugs (10.6% / 5.9% / 5.6%) than for cannabis (8.6% / 3.7% / 2.8%), stimulating illicit drugs (3.8% / 1.3% / 0.6%) and stimulating prescription drugs (2.2% / 0.8% / 0.3%).

### Reasons and goals for PNE

**Fig. 1:**
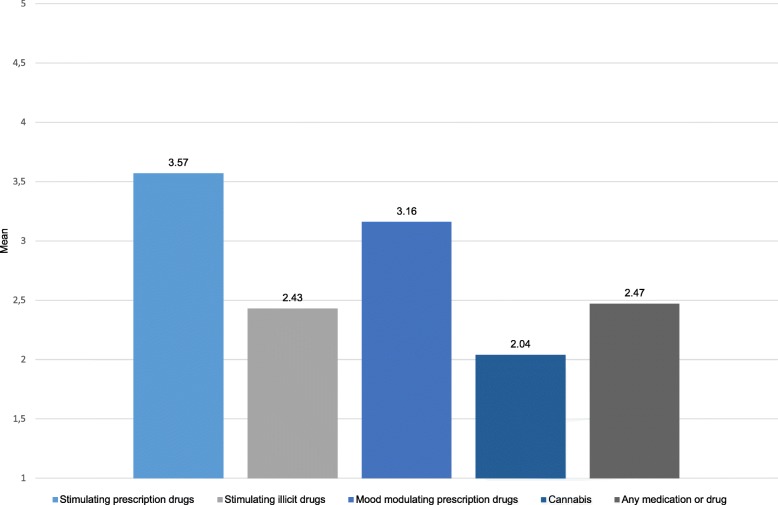
Coping with stressful situations as a reason for the substance intake. Mean scores in the item “What was the reason for the intake? To cope with stressful situations.” Likert scale average (1- I totally disagree to 5 – I totally agree). Stimulating prescription drugs: use of prescription drugs containing amphetamines, methylphenidate, modafinil and/or anti-dementia drugs; Stimulating illicit drugs: use of cocaine, amphetamines and/ or meth-amphetamines; Mood modulating prescription drugs: use of anti-depressants, beta blocker and/ or benzodiazepines); Cannabis: use of cannabis; Any drug: use of any of the listed substances

Coping with stressful situations was a relevant motive for the intake of substances for PNE, but this varied between substance groups. Users of prescription drugs agreed to a higher extent that stress coping was a reason for their use (stimulating prescription drugs: M = 3.57, SD = 1.39; mood modulating prescription drugs: M = 3.16, SD = 1.65) than users of illicit drugs (stimulating illicit drugs M = 2.43, SD = 1.52; cannabis: M = 2.04, SD = 1.43) (see Fig. 1). On the other hand, users of illicit drugs agreed to a higher extent that their goal for the intake was to improve mood (stimulating illicit drugs M = 4.26, SD = 1.16; cannabis: M = 4.12, SD = 1.28) compared to users of prescription drugs (stimulating prescription drugs: M = 3.32, SD = 1.44; mood modulating prescription drugs: M = 3.23, SD = 1.66).

### Stress, the ability to recover from stress and resilience factors in users and non-users of substances for PNE

The ability to recover from stress and the level of perceived stress differed between users (*n* = 435) and non-users (*n* = 686) of any prescription or illicit drug (see Additional file 1): Users had a lower ability to recover from stress than non-users as measured by the Brief Resilience Scale (BRS) (user: M = 3.19, SD = .96; non-user: M = 3.46, SD = .93; *p* < 0.001). This difference was also shown in the subgroups ‘stimulating prescription drugs’ and ‘mood modulating drugs’ but not in the subgroups ‘stimulating illicit drugs’ or ‘cannabis’ (see Additional file 1). Users also reported more perceived stress than non-users as measured by the Perceived Stress Scale (PSS-4) (user: M = 7.46, SD = 2.87; non-user: M = 6.70, SD = 2.79, *p* < .01). The same results were found for all subgroups of PNE users (i.e., stimulating prescription drugs, stimulating illicit drugs, mood modulating drugs and cannabis) (see Additional file 1). Furthermore, compared to non-users, users showed lower values in the three resilience factors. As indicated by the mean scores in the Optimism-Pessimism-2 Scale (SOP-2), users were less optimistic than non-users (user: M = 4.83, SD = 1.21; non-user: M = 5.08, SD = 1.19, *p* < .01) overall as well as in all subgroups of PNE users (see Additional file 1).

In addition, users showed lower self-efficacy beliefs than non-users (user: M = 3.89, SD = .70; non-user: M = 4.02, SD = .72, *p* < .01), as assessed by the Short Scale for Measuring General Self-efficacy Beliefs (ASKU). This result was found in all subgroups of PNE users (see Additional file 1). Furthermore, compared to non-users, users had lower internal locus of control (user: M = 4.11, SD = .74; non-user: M = 4.23, SD = .69, *p* < .05) and higher external locus of control (user: M = 2.59, SD = .83; non-user: M = 2.38, SD = .82, *p* < .01), as measured by the Short Scale for the Assessment of Locus of Control (IE). For internal locus of control, the same difference was only identified for the subgroups ‘mood modulating drugs’ and ‘cannabis’ but not for the subgroups ‘stimulating prescription drugs’ and ‘stimulating illicit drugs’ (see Additional file 1). External locus of control was higher in users compared to non-users in all subgroups (Additional file 1).

### Stress, the ability to recover from stress and resilience factors as factors explaining substance use for PNE

Prior to multivariate logistic regression analyses, we examined the correlations between predictor variables (BRS, PSS-4, ASKU, SOP-2, IE) to assess for potential multicollinearity (Additional file 2). We found medium to high correlations between the predictor variables (range: −.39 to .62). However, the correlations were below the recommended cut-off (.80) indicating that these variables all assess different constructs and multicollinearity can be excluded. The VIF is > 10 which is below the recommended cut-off providing additional evidence against multicollinearity.

Table 3 shows the first and the last step of the adjusted multivariate logistic regression model for mood modulating prescription drugs. In that subgroup, only the BRS remained as predictor in the final model (step 6). Thus, the ability to recover from stress, but not the other predictors was the main factor explaining the use of mood modulation prescription drugs. Each additional unit on the BRS (i.e. the increasing ability to recover from stress) decreases the likelihood for the use of mood modulating prescription drugs by 38% (OR: .62; 95% CI: .47–.81) (see Table 3). The final model explained 36% of the variance (R^2^: .36) (3).

**Table 3:** Adjusted multivariate model of factors associated with use of mood modulating prescription drugs

Variables	step 1^a^				step 6^a^			
	*Coeff. (SE)*	*OR (95% CI)*	*p*	*VIF*	*Coeff. (SE)*	*OR (95% CI)*	*p*	*R* ^*2b*^
BRS	−.43 (.18)	.65 (.46–.93)	.02	2.02	−.48 (.14)	.62 (.47–.81)	<.001	.36
PSS-4	−.12 (.18)	.88 (.63–1.25)	.48	1.77	–	–	–	
SOP-2	−.09 (.17)	.91 (.65–1.28)	.60	1.70	–	–	–	
ASKU	.04 (.20)	1.04 (.71–1.52)	.84	1.72	–	–	–	
IE-I	.09 (.18)	1.09 (.77–1.55)	.63	1.63	–	–	–	
IE-E	.27 (.18)	1.31 (.92–1.86)	.14	1.60	–	–	–	
Sex:								
Male	Reference			1.15	Reference			
Female	−.31 (.29)	.73 (.42–1.29)	.28		–	–	–	
Age	.54 (.20)	1.72 (1.16–2.55)	.01	1.11	.53 (.20)	1.70 (1.15–2.50)	.01	
Education:								
No formal degree	Reference			1.11	Reference			
Secondary modern school	−.73 (1.45)	.48 (.028–8.29)	.61		–	–	–	
Middle school	−.17 (.35)	.84 (.42–.1.69)	.63		–	–	–	
University-entrance diploma	−.16 (.42)	.85 (.38–1.93)	.70		–	–	–	
University degree	−.17 (.45)	.84 (.35–2.02)	.70		–	–	–	
Current or last professional position:								
Skilled worker	Reference			1.05	Reference			
Executive employee	.12 (.57)	1.13 (.37–3.44)	.83		–	–	–	
Non-executive employee	.46 (.46)	1.58 (.64–3.92)	.32		–	–	–	
Civil servants	−.33 (.77)	.72 (.16–.3.24)	.67		–	–	–	
Self-employed	.78 (.64)	2.17 (.62–7.63)	.23		–	–	–	
Other	.13 (.57)	1.14 (.37–3.46)	.82		–	–	–	
Place of residence:								
North Rhine-Westphalia	Reference			1.07	Reference			
Hamburg	.55 (.78)	1.73 (.37–7.98)	.48		–	–	–	
Lower Saxony	.09 (.50)	1.09 (.41–2.90)	.85		–	–	–	
Bremen	.47 (1.77)	1.60 (.05–51.41)	.79		–	–	–	
Schleswig Holstein	.27 (.71)	1.32 (.33–5.24)	.70		–	–	–	
Hesse	−.24 (.56)	.78 (.26–2.35)	.78		–	–	–	
Rhineland-Palatine	.30 (.68)	1.35 (.36–5.07)	.66		–	–	–	
Baden-Wuerttemberg	.16 (.43)	1.17 (.50–2.75)	.71		–	–	–	
Bavaria	−.13 (.43)	.88 (.38–2.03)	.77		–	–	–	
Saarland	−.1.73 (1.49)	.18 (.01–3.30)	.25		–	–	–	
Berlin	−.56 (.78)	.57 (.12–2.63)	.47		–	–	–	
Brandenburg	1.18 (.76)	3.24 (.71–14.81)	.13		–	–	–	
Mecklenburg-Western Pomerania	n. a.	n. a.	n. a.	n. a.	–	–	–	
Saxony	−1.07 (.77)	.35 (.08–1.56)	.17		–	–	–	
Saxony-Anhalt	−1.25 (.89)	.29 (.05–1.65)	.16		–	–	–	
Thuringia	−2.69 (1.34)	.07 (.005–.94)	.05		–	–	–	
Soft enhancer intake:								
No	Reference			1.06	Reference			
Yes	2.29 (.35)	9.89 (5.02–19.51)	<.001		2.25 (.34)	9.49 (4.86–18.52)	.62	

Logistic regression with backward elimination of factors associated with use of mood modulating prescription drugs (users *n* = 114; non-users = 686). First step and final step. n.a. = no data available

^a^Step 1 (full model) and step 6 (reduced model) in multivariate logistic regression using backward variable selection. Predictors were z-standardized before being included in regression analysis. Sociodemographic variables significant in the analyses of mean differences (sex, age, education, current or last professional position, size place of residence, soft enhancer intake) were included as a block in multivariate backward logistic regression. Results are weighted according to the distribution of the general population in Germany as reported by the German office of national statistics. ^b^Nagelkerkes R^2^ in step 6 of stepwise backward selection; *Coeff.* standardised regression coefficient, *SE* standard error, *OR* odds ratio, *CI* confidence interval, *p p* value, *VIF* variance inflation factor (based on multivariate linear regression), *BRS* Brief Resilience Scale, *PSS-4* Perceived Stress Scale, *SOP-2* Optimism-Pessimism-2 Scale, *ASKU* Short Scale for Measuring General Self-efficacy Beliefs, *IE-I* Short Scale for the Assessment of Locus of Control, internal control beliefs; IE-E: Short Scale for the Assessment of Locus of Control, external control beliefs

Table 4 shows the first and the last step of the adjusted multivariate logistic regression model for users of stimulating illicit drugs compared to non-users. Only the degree of optimism as measured by the SOP-2 remained as predictor in the final model (step 6). This means that the resilience factor optimism was the main factor explaining stimulating illicit drug use, but not the ability to recover from stress, perceived stress level or the other resilience factors. Here, each additional unit on the SOP-2 (i.e. the increasing level of optimism of the subject) decreased the likelihood for the use of stimulating illicit drugs compared to non-use by 37% (OR: .63; 95% CI: .47–.86). The final model explained 45% of the variance (R^2^: .45) (4).

**Table 4:** Adjusted multivariate model of factors associated with use of stimulating illicit drugs

Variables	step 1^a^				step 6^a^			
	*Coeff. (SE)*	*OR (95% CI)*	*p*	*VIF*	*Coeff. (SE)*	*OR (95% CI)*	*p*	*R* ^*2b*^
BRS	−.14 (.24)	.87 (.55–1.40)	.57	1.99	–	–	–	.45
PSS-4	−.01 (.23)	1.00 (.64–1.55)	.99	1.79	–	–	–	
SOP-2	−.34 (.21)	.71 (.48–1.07)	.10	1.72	−.46 (.16)	.63 (.47–.86)	<.01	
ASKU	−.11 (.25)	.89 (.55–1.45)	.65	1.71	–	–	–	
IE-I	.07 (.22)	1.07 (.69–1.66)	.77	1.61	–	–	–	
IE-E	.13 (.24)	1.13 (.71–1.80)	.60	1.63	–	–	–	
Sex:								
Male	Reference			1.14	Reference			
Female	−1.17 (.36)	.31 (.15–.63)	.001		− 1.11 (.35)	.33 (.17–.66)	<.01	
Age	−.90 (.26)	.41 (.24–.68)	.001	1.19	−.92 (.26)	.40 (.24–.66)	<.001	
Education:								
No formal degree	Reference			1.15	Reference			
Secondary modern school	2.80 (1.24)	16.48 (1.45–187.61)	.02		2.65 (1.22)	14.09 (1.30–152.86)	.3	
Middle school	−.12 (.43)	.89 (.38–2.07)	.78		–	–	–	
University-entrance diploma	−.62 (.51)	.54 (.20–1.45)	.22		–	–	–	
University degree	−.85 (.66)	.43 (.12–1.57)	.20		–	–	–	
Current or last professional position:								
Skilled worker	Reference			1.06	Reference			
Executive employee	−.07 (.71)	.93 (.23–3.70)	.92		–	–	–	
Non-executive employee	.73 (.51)	2.07 (.76–5.63)	.15		–	–	–	
Civil servants	−.07 (1.08)	.93 (.22–7.74)	.95		–	–	–	
Self-employed	.78 (.84)	2.17 (.42–11.2)	.35		–	–	–	
Other	.39 (.61)	1.47 (.44–4.89)	.53		–	–	–	
Place of residence:								
North Rhine-Westphalia	Reference			1.10	Reference			
Hamburg	2.20 (.95)	9.00 (1.39–58.06)	.02		2.14 (.94)	8.53 (1.37–53.35)	.02	
Lower Saxony	−.11 (.749	.89 (.21–3.83)	.89		–	–	–	
Bremen	3.35 (1.46)	28.36 (1.64–491.68)	.02		3.22 (1.43)	25.08 (1.53–411.61)	.02	
Schleswig Holstein	−.37 (1.10)	.69 (.08–5.91)	.74		–	–	–	
Hesse	1.00 (.62)	2.72 (.80–9.23)	.11		–	–	–	
Rhineland-Palatine	.82 (1.00)	2.27 (.32–16.04)	.41		–	–	–	
Baden-Wuerttemberg	.66 (.56)	1.93 (.64–5.79)	.24		–	–	–	
Bavaria	.004 (.612)	1.00 (.30–3.33)	.99		–	–	–	
Saarland	n.a.	n.a.	n.a.	n.a.	n.a.	n.a.	n.a.	
Berlin	1.66 (7.43)	5.28 (1.23–22.66)	.03		1.53 (.73)	4.62 (1.11–19.27)	.04	
Brandenburg	2.04 (1.13)	7.71 (.84–70.71)	.07		–	–	–	
Mecklenburg-Western Pomerania	.86 (.86)	2.36 (.44–12.83)	.32		–	–	–	
Saxony	.62 (.77)	1.85 (.41–8.36)	.32		–	–	–	
Saxony-Anhalt	−1.43 (1.42)	.24 (.02–3.89)	.31		–	–	–	
Thuringia	.02 (1.16)	1.02 (.11–9.9)	.99		–	–	–	
Soft enhancer intake:								
No	Reference			1.12	Reference			
Yes	3.02 (.60)	20.39 (6.35–65.52)	<.001		3.03 (.60)	20.73 (6.46–66.47)	<.001	

Logistic regression with backward elimination of factors associated with use of stimulating illicit drugs (users *n* = 114; non-users = 686). First step and final step. n.a. = no data available

Further notes see Table 3

Table 5 shows the first and the last step of the adjusted multivariate logistic regression model for users of cannabis compared to non-users. Here again, only the degree of optimism as measured by the SOP-2 remained as predictor in the final model (step 6) meaning that the resilience factor optimism, but not the ability to recover from stress, the perceived stress level or the other resilience factors were main factors explaining the use of cannabis.

**Table 5:** Adjusted multivariate model of factors associated with use of cannabis

Variables	step 1^a^				step 6^a^			
	*Coeff. (SE)*	*OR (95% CI)*	*p*	*VIF*	*Coeff. (SE)*	*OR (95% CI)*	*p*	*R* ^*2b*^
BRS	−.13 (.16)	.88 (.64–1.21)	.44	1.96	–	–	–	.33
PSS-4	−.06 (.15)	.94 (.70–1.27)	.68	1.75	–	–	–	
SOP-2	−.14 (.15)	.87 (.66–1.16)	.34	1.68	−.31 (.11)	.74 (.59–.92)	.01	
ASKU	−.11 (.17)	.89 (.64–1.25)	.51	1.73	–	–	–	
IE-I	−.12 (.15)	.89 (.66–1.12)	.44	1.65	–	–	–	
IE-E	.14 (.15)	1.15 (.86–1.54)	.34	1.54	–	–	–	
Sex:								
Male	Reference			1.11	Reference			
Female	−.67 (.24)	.51 (.32–.82)	.51		−.63 (.23)	.53 (.34–.84)	.01	
Age	−.69 (.17)	.50 (.36–.70).	<.001	1.14	−.68 (.17)	.50 (.36–.70)	<.001	
Education:								
No formal degree	Reference			1.16	Reference			
Secondary modern school	1.68 (.96)	5.38 (.81–35.62)	.08		–	–	–	
Middle school	−.02 (.32)	.99 (.53–1.82)	.96		–	–	–	
University-entrance diploma	−.14 (.37)	.87 (.42–1.80)	.71		–	–	–	
University degree	.60 (.39)	1.82 (.85–3.90)	.12		–	–	–	
Current or last professional position:								
Skilled worker	Reference				Reference			
Executive employee	−.49 (.47)	.61 (.24–1.53)	.29	1.05	–	–	–	
Non-executive employee	.05 (.36)	1.05 (.52–2.14)	.89		–	–	–	
Civil servants	−.10 (.59)	.91 (.29–2.88)	.87		–	–	–	
Self-employed	−.29 (.60)	.75 (.23–2.43)	.63		–	–	–	
Other	.15 (.43)	1.16 (.50–2.70)	.73		–	–	–	
Place of residence:								
North Rhine-Westphalia	Reference			1.05	Reference			
Hamburg	.94 (.69)	2.56 (.67–9.77)	.17		–	–	–	
Lower Saxony	.01 (.45)	1.01 (.42–2.43)	.98		–	–	–	
Bremen	.29 (1.15)	1.34 (.14–12.60)	.80		–	–	–	
Schleswig Holstein	.01 (.71)	1.01 (.25–4.07)	.99		–	–	–	
Hesse	.71 (.43)	2.04 (.87–4.74)	.10		–	–	–	
Rhineland-Palatine	1.11 (.59)	3.04 (.95–9.74)	.06		–	–	–	
Baden-Wuerttemberg	.66 (.39)	1.92 (.89–4.15)	.10		–	–	–	
Bavaria	.34 (.38)	1.40 (.67–2.95)	.37		–	–	–	
Saarland	−1.62 (1.06)	.20 (.03–1.58)	.13		–	–	–	
Berlin	.62 (.55)	1.86 (.63–5.49)	.26		–	–	–	
Brandenburg	1.63 (.72)	5.10 (1.24–20.92)	.03		–	–	–	
Mecklenburg-Western Pomerania	.31 (.73)	1.36 (.32–5.72)	.68		–	–	–	
Saxony	−.03 (.55)	1.03 (.35–3.00)	.96		–	–	–	
Saxony-Anhalt	−1.87 (1.03)	.16 (.02–1.17)	.07		–	–	–	
Thuringia	−.17 (.74)	.84 (.20–3.61)	.82		–	–	–	
Soft enhancer intake:								
No	Reference			1.09	Reference			
Yes	1.79 (.26)	5.96 (3.55–10.01)	<.001		1.80 (.26)	6.05 (3.63–10.08)	<.001	

Logistic regression with backward elimination of factors associated with use of cannabis (users *n* = 260; non-users = 686). First step and final step. n.a. = no data available

Further notes see Table 3

Overall, each additional unit on the SOP-2 (i.e. the increasing level of optimism of the subject) decreases the likelihood of cannabis use by 26% (OR: .74; 95% CI: .59–.92). The final model explained 33% of the variance (R^2^: .33) (Table 5).

Table 6 shows the first and the last step of the adjusted multivariate logistic regression model for users of stimulating prescription drugs compared to non-users. Only the level of perceived stress as measured by the PSS-4 remained as predictor in the final model (step 6). This means that perceived stress, but not the ability to recover from stress or any of the resilience factors was the main factor explaining stimulating prescription drug use. Each unit on the PSS-4 (i.e. the increasing level of perceived stress) increases the likelihood for the use of stimulating prescription drugs almost three times (OR: 2.89; 95% CI: 1.49–5.46). The final model explains 45% (R^2^: .45) of variance in that subgroup (Table 6).

**Table 6:** Adjusted multivariate model of factors associated with use of stimulating prescription drugs

Variables	step 1^a^				step 6^a^			
	*Coeff. (SE)*	*OR (95% CI)*	*p*	*VIF*	*Coeff. (SE)*	*OR (95% CI)*	*p*	*R* ^*2b*^
BRS	−.72 (.42)	.49 (.21–1.12)	.09	2.11	–	–	–	.45
PSS-4	.78 (.43)	2.19 (.95–5.05)	.07	1.75	1.05 (.33)	2.86 (1.49–5.46)	<.01	
SOP-2	.01 (.38)	1.00 (.48–2.13)	.98	1.76	–	–	–	
ASKU	.07 (.5)	1.07 (.40–2.82)	.90	1.76	–	–	–	
IE-I	.18 (.43)	1.19 (.52–2.74)	.68	1.66	–	–	–	
IE-E	−.16 (.38)	.86 (.40–1.81)	.68	1.60	–	–	–	
Sex:								
Male	Reference			1.15	Reference			
Female	−.15 (.60)	.86 (.27–2.76)	.80		–	–	–	
Age	−.89 (.47)	.41 (.17–1.03)	.06	1.21	.53 (.20)	1.70 (1.15–2.50)	<.01	
Education:								
No formal degree	Reference			1.11	Reference			
Secondary modern school	n.a.	n.a.	n.a.		–	–	–	
Middle school	−1.21 (.83)	.30 (.06–1.53)	.15		–	–	–	
University-entrance diploma	.07 (.81)	1.08 (.22–5.28).	.93		–	–	–	
University degree	−.61 (1.09)	.54 (.06–4.61)	.57		–	–	–	
Current or last professional position:								
Skilled worker	Reference				Reference			
Executive employee	2.16 (1.25)	8.69 (.75–100.30)	.08	1.05	–	–	–	
Non-executive employee	1.00 (1.15)	2.72 (.29–25.62)	.38		–	–	–	
Civil servants	1.78 (1.75)	5.92 (.19–181.33)	.31		–	–	–	
Self-employed	2.74 (1.41)	15.44 (.98–242.95)	.05		–	–	–	
Other	1.76 (1.42)	5.81 (.36–94.30)	.22		–	–	–	
Place of residence:								
North Rhine-Westphalia	Reference			1.10	Reference			
Hamburg	.07 (1.55)	1.07 (0.05–22.47)	.96		–	–	–	
Lower Saxony	−1.67 (1.59)	.19 (.01–4.26)	.29		–	–	–	
Bremen	n.a.	n.a.	n.a.		–	–	–	
Schleswig Holstein	.17 (1.53)	1.18 (.06–23.60)	.91		–	–	–	
Hesse	.10 (1.09)	1.10 (.13–9.27)	.93		–	–	–	
Rhineland-Palatine	.70 (1.38)	2.01 (.13–30.15)	.61		–	–	–	
Baden-Wuerttemberg	.51 (.86)	1.66 (.31–9.03)	.56		–	–	–	
Bavaria	−.61 (.97)	.54 (.08–3.63)	.53		–	–	–	
Saarland	n.a.	n.a.	n.a.		–	–	–	
Berlin	n.a.	n.a.	n.a.		–	–	–	
Brandenburg	1.389 (2.18	3.98 (.06–286.58)	.53		–	–	–	
Mecklenburg-Western Pomerania	n.a.	n.a.	n.a.		–	–	–	
Saxony	−.09 (1.23)	.92 (.08–10.23)	.94		–	–	–	
Saxony-Anhalt	n.a.	n.a.	n.a.		–	–	–	
Thuringia	n.a.	n.a.	n.a.		−2.62 (1.32)	.07 (.01–.97)	.05	
Soft enhancer intake:								
No	Reference			1.09	Reference			
Yes	2.63 (1.12)	13.83 (1.55–123.54)	.02		2.25 (.34)	9.49 (4.86–18.52)	<.001	

Logistic regression with backward elimination) of factors associated with use of stimulating prescription drugs (users *n* = 48; non-users = 686). First step and final step. n.a. = no data available

Further notes see Table 3

## Discussion

This is the first study providing data on the use of prescription and illicit drugs for PNE in a representative sample in Germany. In addition, it contributes to previous studies in the field of PNE use by investigating the relationship between PNE use and the ability to recover from stress for the first time.

We found a lifetime prevalence rate of any substance use for PNE of 38.8% which was higher compared to previous studies (1–20%) [4, 7, 16–20] . There are several possible explanations for the higher prevalence rates in our survey compared to other studies. First of all, we used a broader definition of PNE which referred not only to PNE use for enhancing cognitive performance but also to improving mood or reducing nervousness without medical indication. This broader definition may explain the higher prevalence rates found in this study by including more users with different motives for the substance intake. In this study, we decided to use a broader definition of PNE since it refers not only to the objective of achieving higher cognitive performance, but also to reaching a mental state which allows coping with daily tasks. In contrast, previous studies only assessed the intake of prescription stimulants [4, 13, 19] or prescription medication [1].

Second, the use of psychoactive substances varies between different study populations. Previous studies were limited to specific groups such as students or pupils, whereas our survey delivers representative data for the German adult population.

Third, by using a closed envelope technique for sensitive questions, we provided a high degree of confidentiality for the respondents which may have resulted in a higher and more reliable prevalence rate compared to studies using less anonymous techniques. Indeed, previous studies providing a higher degree of confidentiality, such as online surveys or surveys using the randomized response technique, revealed higher prevalence rates with respect to sensitive questions as compared to standard, non-anonymous surveys [7, 15, 16].

At first sight, cannabis seems to play the most important role for PNE in our survey as we found the highest lifetime prevalence (23%) compared to other substance groups. However, it is likely that the majority of cannabis users took the substance many years ago only for a short period of time since the last year prevalence already declined to 9%. Last week prevalence (which can be considered as an indicator for current regular use) was only 3% for cannabis. In comparison, mood modulating prescription drugs seem to play a more important role in everyday life as we found a last week prevalence of 6%.

A major goal of this study was to assess the relationship of substance use and perceived stress, the ability to cope with stress and three resilience factors (self-efficacy, locus of control and optimism [37, 38]). The individual ability to recover from stress (as measured by the BRS) was associated with a lower risk of mood modulating drug use. Furthermore, subjects reporting high perceived stress (as measured by the PSS-4) were more likely to use stimulating prescription drugs such as methylphenidate, modafinil or amphetamines. These results provide evidence for a slightly differential use of stimulating prescription drugs and mood modulating drugs. With regard to the effect sizes, the adjusted multivariate models used explain 36% of the variance for the use of mood modulating prescription drugs and 45% of the variance for the use of stimulating prescription drugs. Regarding the predictor variables we found clinical meaningful effects. The OR for each additional unit on the BRS was .62 (a decrease of 38%) for mood modulating prescription drugs with a 95% CI of .47 to .81 (53% to 19%). This means that there is a 95% probability that the population parameter lies within the interval, when considering the lower CI that would result in a decrease in the likelihood of 19%. The OR for each additional unit on the PSS-4 was 2.89 (an almost threefold increase) for stimulating prescription drugs with a 95% CI of 1.49 to 5.46 (a 1.5 to 5.5-fold increase), which means that when considering the lower CI the likelihood would still be 50% higher. Our results are also in line with previous research on the effect of risk factors on PNE use. In a previous study on surgeons [16], we found that ORs that were lower or in a similar range (e.g., ‘pressure to perform at work’: OR 1.29, 95% CI: 1.00 to 1.67; ‘gross income’: OR 1.34, 95% CI: 1.09 to 1.64).

But nevertheless the use of both substance groups is associated with “stress” in a broader context, as both user-groups named “stress coping” as an important goal for the substance use.

Neither the ability to recover from stress nor perceived stress were associated with the use of stimulating illicit drugs or cannabis in this study. However, in a previous study among German students, participants using cannabis for the purpose of cognitive enhancement reported more stress than non-users as they perceived the pressure to perform as more burdening [48]. Since these analyses referred to different stress measurements and were not controlled for other factors (as in our multivariate model), it is not possible to directly compare the results. We also identified an association between the use of stimulating illicit drugs or cannabis and a higher level of pessimism. This is in line with previous studies describing pessimism as a risk factor of illicit drug consumption [49].

Being pessimistic could be connected with the goal to improve mood which was a more important goal for the use of illicit than for prescription drugs. This could indicate different patterns of substance use: prescription drugs are rather used goal-oriented as an instrument in stressful situations as a coping strategy whereas illicit drugs are rather used with more general underlying goals such as improving mood. This corresponds with results of a study that indicates that stress coping is a more prevalent goal for the use of prescription medication than for the use of illicit drugs. This study also showed that improving mood measured by the goal “euphoria” seems to be more important for the use of illicit drugs than for prescription medication [50]. We did not find an association of any substance use for PNE with the degree of self-efficacy as was described in an earlier study [51].

This study has several limitations. First, we only assessed adults over 18 years of age which does not allow any conclusions for younger people that were identified as an important at risk population for PNE use [52].

Second, as we conducted the survey with a quota and not a probability sample, our data can only be generalized to the population in Germany, but not to other countries.

Third, in this study we only used the BRS as proxy measure for resilience as outcome by assessing the ability to recover from stress. To date, the BRS is the only scale that was developed based on an outcome definition of resilience [53]. Other existing resilience scales are either based on a trait-oriented approach (e.g., Dispositional Resilience Scale [DRS]) [54]or focus on measuring the availability of resources and protective factors to maintain or regain mental health despite significant adversities (e.g., Connor-Davidson Resilience Scale [CD-RISC]) [55]. To assess resilience as defined (i.e., mental health despite stress), the individual stressor exposure should also be considered when measuring resilience, as suggested by Kalisch and colleagues [56]. As this was not possible in the current study, future representative surveys on resilience and PNE use are required.

Fourth, we did not adjust for multiple testing which may increase the risk of type 1 error inflation. However, our main results regarding the relationship of substance use and perceived stress, the ability to cope with stress and three resilience factors, the respective *p*-values of the odds ratios are between *p* = .006 and *p* < .001. We therefore infer that our main results are robust and not affected by alpha inflation.

Fifth, data were collected cross-sectionally in this study. As a consequence, the analyses are only explorative and causal inferences about the associations found cannot be drawn. The effects of low ability to recover from stress and high perceived stress on PNE use and the potential positive effects of PNE use on these variables cannot be disentangled. This limits the conclusions in this study and underlines the importance of conducting longitudinal studies in this field.

## Conclusions

Based on the results of this study, interventions fostering the ability to recover from stress and reducing perceived stress could have the potential to prevent PNE use at an early stage. We are currently investigating the evidence base (randomized controlled trials) of resilience trainings available so far [57] and have discussed a methodological framework of the suitable design of resilience trainings [58]. In the future, randomized controlled intervention studies to support the individual ability to recover from stress have to be performed in order to demonstrate that PNE use can be reduced in at risk populations.

In sum, our results provide evidence that PNE is not only used to enhance cognitive performance, improve mood or reduce nervousness, but also to cope with stress. PNE as a strategy for stress management and measures to prevent its intake should be further investigated.

## Additional files

Additional file 1: Sample characteristics per substance group. (DOCX 70 kb)

Additional file 2: Correlations between the ability to recover from stress, perception of stress and resilience factors. (DOCX 27 kb)

## Abbreviations

ASKU: Short Scale for Measuring General Self-Efficacy Beliefs
BRS: Brief Resilience Scale
CI: Confidence intervall
Coeff: Standardised regression coefficient
IE-E: Short Scale for the Assessment of Locus of Control, external control beliefs.
IE-I: Short Scale for the Assessment of Locus of Control, internal control beliefs.
M: Mean
OR: Odds ratio
PNE: Pharmacological neuroenhancement
PSS-4: Perceived Stress Scale
SD: Standard deviation
SE: standard error
SOP-2: Optimism-Pessimism-2 Scale
VIF: Variance inflation factor
