# Supplementary material for: Pharmacological neuroenhancement and the ability to recover from stress – a representative cross-sectional survey among the German population
Source: Subst Abuse Treat Prev Policy. 2018 Oct 22;13:37. doi: 10.1186/s13011-018-0174-1 (PMC6198480; doi:10.1186/s13011-018-0174-1)
Supplement: Supplementary file 1 — Sample characteristics per substance group. (DOCX 70 kb) [file 13011_2018_174_MOESM1_ESM.docx]

Additional file 1: Sample characteristics per substance group

|  | **Any medication or drug (n=435)** | | | **Stimulating prescription drugs (n=48)** | | | **Stimulating illicit drugs (n=114)** | | | **Mood modulating drugs (n=225)** | | | **Cannabis (n=260)** | | | **Non-Users (N=686)** | | |
| --- | --- | --- | --- | --- | --- | --- | --- | --- | --- | --- | --- | --- | --- | --- | --- | --- | --- | --- |
|  | **M** | **95% CI** |  | **M** | **95% CI** |  | **M** | **95% CI** |  | **M** | **95% CI** |  | **M** | **95% CI** |  | **M** | **95% CI** |  |
|  |  | min | max |  | min | max |  | min | max |  | min | max |  | min | max |  | min | max |
| **Age** | 47.66* | 45.97 | 49.34 | 43.62* | 37.81 | 49.44 | 38.01* | 35.30 | 40.71 | 55.45 | 53.30 | 57.59 | 41.21* | 39.31 | 43.12 | 53.39 | 52.03 | 54.75 |
|  | **% valid (n)** | **95% CI** |  | **% valid (n)** | **95% CI** |  | **% valid (n)** | **95% CI** |  | **% valid (n)** | **95% CI** |  | **% valid (n)** | **95% CI** |  | **% valid (n)** | **95% CI** |  |
|  |  | min | max |  |  |  |  | min | max |  | min | max |  | min | max |  | min | max |
| **Sex** |  |  |  |  |  |  | * |  |  |  |  |  | * |  |  |  |  |  |
| Male | 51.72 (225) | 47.03 | 56.42 | 43.75 (21) | 29.72 | 57.78 | 66.96 (77) | 58.36 | 75.55 | 44.44 (100) | 37.95 | 50.94 | 61.54 (160) | 55.62 | 67.45 | 46.00 (316) | 42.27 | 49.72 |
| Female | 48.28(210) | 43.58 | 52.97 | 56.25 (27) | 42.22 | 70.28 | 33.04 (38) | 24.45 | 41.64 | 55.56 (125) | 49.06 | 62.05 | 38.46 (100) | 32.55 | 44.38 | 54.00 (371) | 50.28 | 57.73 |
|  |  |  |  |  |  |  |  |  |  |  |  |  |  |  |  |  |  |  |
| **Education** |  |  |  |  |  |  |  |  |  |  |  |  |  |  |  |  |  |  |
| No formal degree | 1.41 (6) | 0.29 | 2.53 | - | - | - | 4.50 (5) | 0.65 | 8.36 | 1.82 (4) | 0.05 | 3.58 | 2.35 (6) | 0.49 | 4.21 | 1.61 (11) | 0.67 | 2.55 |
| Secondary modern school^a^ | 31.29 (133) | 26.89 | 35.70 | 32.61 (15) | 19.06 | 46.16 | 28.83 (32) | 20.40 | 37.26 | 34.09 (75) | 27.83 | 40.35 | 25.10 (64) | 19.78 | 30.42 | 33.24 (227) | 29.70 | 36.77 |
| Middle school | 30.35 (129) | 25.98 | 34.72 | 19.57 (9) | 8.10 | 31.03 | 33.33 (37) | 24.56 | 42.10 | 31.82 (70) | 25.66 | 37.97 | 29.80 (76) | 24.19 | 35.42 | 30.89 (211) | 27.43 | 34.36 |
| University-entrance diploma^c^ | 17.65 (75) | 14.02 | 21.27 | 30.43 (14) | 17.14 | 43.73 | 23.42 (26) | 15.54 | 31.30 | 15.91 (35) | 11.08 | 20.74 | 21.18 (54) | 16.16 | 26.19 | 17.72 (121) | 14.85 | 20.58 |
| University degree | 19.29 (82) | 15.54 | 23.05 | 17.39 (8) | 6.44 | 28.34 | 9.91 (11) | 4.35 | 15.47 | 16.36 (36) | 11.48 | 21.25 | 21.57 (55) | 16.52 | 26.62 | 16.54 (113) | 13.76 | 19.33 |
| **Current or last professional position** |  |  |  |  |  |  |  |  |  | * |  |  |  |  |  |  |  |  |
| Skilled worker | 14.35 (62) | 11.05 | 17.66 | 4.26 (2) | 0.0 | 10.03 | 14.16 (16) | 7.73 | 20.59 | 10.81 (24) | 6.73 | 14.90 | 13.90 (36) | 9.69 | 18.11 | 14.22 (97) | 11.60 | 16.84 |
| Executive employee | 10.65 (46) | 7.74 | 13.56 | 17.02 (8) | 6.28 | 27.77 | 7.96 (9) | 2.97 | 12.96 | 12.16 (27) | 7.86 | 16.46 | 9.27 (24) | 5.74 | 12.80 | 14.22 (97) | 11.60 | 16.84 |
| Non-executive employee | 43.29 (187) | 38.61 | 47.96 | 34.04 (16) | 20.50 | 47.59 | 40.71 (46) | 31.65 | 49.77 | 45.50 (101) | 38.94 | 52.05 | 42.86 (111) | 36.83 | 48.88 | 43.99 (300) | 40.26 | 47.71 |
| Civil servants | 5.56 (24) | 3.40 | 7.72 | 8.51 (4) | 0.53 | 16.49 | 3.54 (4) | 0.13 | 6.95 | 5.41 (12) | 2.43 | 8.38 | 5.41 (14) | 2.65 | 8.16 | 6.45 (44) | 4.61 | 8.30 |
| Self-employed | 6.02 (26) | 3.78 | 8.26 | 6.38 (3) | 0.0 | 13.37 | 6.19 (7) | 1.75 | 10.64 | 7.66 (17) | 4.16 | 11.16 | 6.18 (16) | 3.25 | 9.11 | 3.37 (23) | 2.02 | 4.73 |
| Other | 14.81 (64) | 11.46 | 18.16 | 19.15 (9) | 7.90 | 30.40 | 21.24 (24) | 13.70 | 28.78 | 16.22 (36) | 11.37 | 21.07 | 15.83 (41) | 11.38 | 20.28 | 13.20 (90) | 10.66 | 15.74 |
| **Shift work** |  |  |  |  |  |  |  |  |  |  |  |  |  |  |  |  |  |  |
| Yes | 15.44 (44) | 11.24 | 19.63 | 10.71 (3) | 0.00 | 22.17 | 19.10 (17) | 10.93 | 27.27 | 12.60 (16) | 6.83 | 18.37 | 14.51 (28) | 9.54 | 19.48 | 19.95 (75) | 15.91 | 23.99 |
| No | 84.56 (241) | 80.37 | 88.76 | 89.29 (25) | 77.83 | 100.00 | 80.90 (72) | 72.73 | 89.07 | 87.40 (111) | 81.63 | 93.17 | 85.49 (165) | 80.52 | 90.46 | 80.05 (301) | 76.01 | 84.09 |
| **Weekly working hours** |  |  |  |  |  |  |  |  |  | * |  |  |  |  |  |  |  |  |
| <20 hours | 6.67 (19) | 3.77 | 9.56 | - | - | - | 4.49 (4) | 0.19 | 8.80 | 12.50 (16) | 6.77 | 18.23 | 3.65 (7) | 0.99 | 6.30 | 4.29 (16) | 2.23 | 6.35 |
| 20-29 | 8.77 (25) | 5.49 | 12.06 | - | - | - | 5.62 (5) | 0.83 | 10.40 | 11.72 (15) | 6.15 | 17.29 | 6.77 (13) | 3.22 | 10.32 | 9.92 (37) | 6.89 | 12.95 |
| 30-40 | 50.53 (144) | 44.72 | 56.33 | 65.38 (17) | 47.10 | 83.67 | 57.30 (51) | 47.03 | 67.58 | 44.53 (57) | 35.92 | 53.14 | 54.17 (104) | 47.12 | 61.21 | 50.40 (188) | 45.33 | 55.48 |
| 41-50 | 24.91 (71) | 19.89 | 29.93 | 23.08 (6) | 6.88 | 39.27 | 22.47 (20) | 13.80 | 31.14 | 21.88 (28) | 14.71 | 29.04 | 26.04 (50) | 19.83 | 32.25 | 25.74 (96) | 21.30 | 30.17 |
| > 50 | 9.12 (26) | 5.78 | 12.47 | 11.54 (3) | 0.00 | 23.82 | 10.11 (9) | 3.85 | 16.38 | 9.38 (12) | 4.33 | 14.42 | 9.38 (18) | 5.25 | 13.50 | 9.65 (36) | 6.65 | 12.65 |
| **Federal State** |  |  |  |  |  |  | * |  |  |  |  |  | * |  |  |  |  |  |
| North Rhine-Westphalia | 22.02 (96) | 18.13 | 25.91 | 28.57 (14) | 15.92 | 41.22 | 24.14 (28) | 16.35 | 31.93 | 25.22 (57) | 19.56 | 30.88 | 20.38 (53) | 15.49 | 25.28 | 20.93 (144) | 17.89 | 23.97 |
| Hamburg | 2.52 (11) | 1.05 | 3.99 | 2.04 (1) | 0.00 | 6.00 | 4.31 (5) | 0.61 | 8.01 | 3.10 (7) | 0.84 | 5.36 | 3.85 (10) | 1.51 | 6.18 | 3.34 (23) | 2.00 | 4.69 |
| Lower Saxony | 9.40 (41) | 6.66 | 12.14 | 4.08 (2) | 0.00 | 9.62 | 5.17 (6) | 1.14 | 9.20 | 9.29 (21) | 5.51 | 13.08 | 8.85 (23) | 5.39 | 12.30 | 9.88 (68) | 7.65 | 12.11 |
| Bremen | 1.15 (5) | 0.15 | 2.15 | - | - | - | 1.72 (2) | 0.00 | 4.09 | 1.33 (3) | 0.00 | 2.82 | 0.77 (2) | 0.00 | 1.83 | 0.73 (5) | 0.09 | 1.36 |
| Schleswig Holstein | 3.67 (16) | 1.90 | 5.43 | 2.04 (1) | 0.00 | 6.00 | 1.72 (2) | 0.00 | 4.09 | 3.98 (9) | 1.43 | 6.53 | 3.08 (8) | 0.98 | 5.18 | 3.78 (26) | 2.35 | 5.20 |
| Hesse | 8.26 (36) | 5.67 | 10.84 | 8.16 (4) | 0.50 | 15.83 | 13.79 (16) | 7.52 | 20.07 | 6.19 (14) | 3.05 | 9.34 | 11.54 (30) | 7.65 | 15.42 | 6.54 (45) | 4.69 | 8.39 |
| Rhineland-Palatine | 5.73 (25) | 3.55 | 7.92 | 12.24 (6) | 3.07 | 21.42 | 5.17 (6) | 1.14 | 9.20 | 5.75 (13) | 2.72 | 8.79 | 5.77 (15) | 2.94 | 8.60 | 4.36 (30) | 2.83 | 5.89 |
| Baden-Wuerttemberg | 13.76 (60) | 10.53 | 17.00 | 10.20 (5) | 1.73 | 18.68 | 11.21 (13) | 5.47 | 16.95 | 13.72 (31) | 9.23 | 18.20 | 13.85 (36) | 9.65 | 18.04 | 11.63 (80) | 9.23 | 14.02 |
| Bavaria | 14.91 (65) | 11.56 | 18.25 | 18.37 (9) | 7.53 | 29.21 | 9.48 (11) | 4.15 | 14.81 | 16.37 (37) | 11.55 | 21.20 | 13.85 (36) | 9.65 | 18.04 | 15.41 (106) | 12.71 | 18.10 |
| Saarland | 0.92 (4) | 0.02 | 1.81 | - | - | - | - | - | - | 1.33 (3) | 0.00 | 2.82 | 0.38 (1) | 0.00 | 1.14 | 1.74 (12) | 0.77 | 2.72 |
| Berlin | 5.28 (23) | 3.18 | 7.37 | 2.04 (1) | 0.00 | 6.00 | 8.62 (10) | 3.51 | 13.73 | 3.54 (8) | 1.13 | 5.95 | 5.77 (15) | 2.94 | 8.60 | 3.34 (23) | 2.00 | 4.69 |
| Brandenburg | 3.44 (15) | 1.73 | 5.15 | 2.04 (1) | 0.00 | 6.00 | 3.45 (4) | 0.13 | 6.77 | 4.42 (10) | 1.74 | 7.11 | 3.08 (8) | 0.98 | 5.18 | 3.34 (23) | 2.00 | 4.69 |
| Mecklenburg-Western Pomerania | 2.06 (9) | 0.73 | 3.40 | 2.04 (1) | 0.00 | 6.00 | 3.45 (4) | 0.13 | 6.77 | 0.88 (2) | 0.00 | 2.11 | 2.31 (6) | 0.48 | 4.13 | 2.62 (18) | 1.42 | 3.81 |
| Saxony | 3.90 (17) | 2.08 | 5.72 | 4.08 (2) | 0.00 | 9.62 | 5.17 (6) | 1.14 | 9.20 | 2.65 (6) | 0.56 | 4.75 | 3.85 (10) | 1.51 | 6.18 | 6.69 (46) | 4.82 | 8.55 |
| Saxony-Anhalt | 1.61 (7) | 0.43 | 2.79 | - | - | - | 1.72 (2) | -0.64 | 4.09 | 1.77 (4) | 0.05 | 3.49 | 1.15 (3) | 0.00 | 2.45 | 3.63 (7) | 2.24 | 5.03 |
| Thuringia | 1.38 (6) | 0.28 | 2.47 | 4.08 (2) | 0.00 | 9.62 | 0.86 (1) | 0.00 | 2.54 | 0.44 (1) | 0.00 | 1.31 | 1.54 (4) | 0.04 | 3.03 | 3.63 (25) | 2.24 | 5.03 |
| **Size place of residence (inhabitants)** | * |  |  |  |  |  | * |  |  | * |  |  | * |  |  |  |  |  |
| < 2.000 | 7.82 (34) | 5.29 | 10.34 | 10.20 (5) | 1.73 | 18.68 | 6.09 (7) | 1.72 | 10.46 | 6.19 (14) | 3.05 | 9.34 | 8.46 (22) | 5.08 | 11.84 | 8.31 (57) | 6.24 | 10.37 |
| 2.000-20.000 | 29.66 (129) | 25.36 | 33.95 | 38.78 (19) | 25.13 | 52.42 | 27.83 (32) | 19.64 | 36.02 | 30.09 (68) | 24.11 | 36.07 | 27.69 (72) | 22.25 | 33.13 | 37.32 (256) | 33.70 | 40.94 |
| 20.000-100.000 | 25.75 (112) | 21.64 | 29.86 | 20.41 (10) | 9.12 | 31.69 | 25.22 (29) | 17.28 | 33.15 | 25.66 (58) | 19.97 | 31.36 | 25.77 (67) | 20.45 | 31.09 | 28.72 (197) | 25.33 | 32.10 |
| > 100.000 | 36.78 (160) | 32.25 | 41.31 | 30.61 (15) | 17.71 | 43.52 | 40.87 (47) | 31.88 | 49.85 | 38.05 (86) | 31.72 | 44.38 | 38.08 (99) | 32.17 | 43.98 | 36.78 (176) | 32.25 | 41.31 |
| **Soft enhancer intake** | 86.31 (372)* | 83.07 | 89.59 | 95.74 (45)* | 89.97 | 100.00 | 95.54 (107)* | 91.71 | 99.36 | 88.79 (198)* | 84.65 | 92.93 | 85.21 (219)* | 80.87 | 89.55 | 49.48 (334) | 45.71 | 53.25 |
|  | **M** | **95% CI** |  | **M** | **95% CI** |  | **M** | **95% CI** |  | **M** | **95% CI** |  | **M** | **95% CI** |  | **M** | **95% CI** |  |
|  |  | min | max |  | min | max |  | min | max |  | min | max |  | min | max |  | min | max |
| **BRS** | 3.19* | 3.10 | 3.28 | 2.92* | 2.62 | 3.23 | 3.28 | 3.10 | 3.46 | 2.98* | 2.85 | 3.11 | 3.33 | 3.21 | 3.44 | 3.46 | 3.39 | 3.53 |
| **PSS-4** | 7.46* | 7.18 | 7.73 | 8.46* | 7.75 | 9.16 | 7.81* | 7.27 | 8.36 | 7.69* | 7.29 | 8.09 | 7.49* | 7.14 | 7.83 | 6.70 | 6.49 | 6.91 |
| **IE - internal** | 4.11* | 4.04 | 4.18 | 4.03 | 3.82 | 4.23 | 4.09 | 3.94 | 4.24 | 4.07* | 3.97 | 4.18 | 4.09* | 4.01 | 4.18 | 4.23 | 4.17 | 4.28 |
| **IE - external** | 2.59* | 0.83 | 2.51 | 2.91* | 2.61 | 3.21 | 2.70* | 2.53 | 2.87 | 2.69* | 0.84 | 2.58 | 2.58* | 2.48 | 2.68 | 2.38 | 2.32 | 2.44 |
| **ASKU** | 3.89* | 3.82 | 3.95 | 3.66* | 3.42 | 3.91 | 3.83* | 3.69 | 3.96 | 3.87* | 3.77 | 3.97 | 3.91* | 3.82 | 3.99 | 4.02 | 3.97 | 4.08 |
| **SOP-2** | 4.83* | 4.71 | 4.94 | 4.46* | 4.08 | 4.83 | 4.65* | 4.41 | 4.88 | 4.65* | 4.49 | 4.80 | 4.90* | 4.75 | 5.04 | 5.08 | 4.99 | 5.17 |

^a^ equivalent to German “Hauptschule” degree after 9 years of formal education. ^b^ equivalent to German “Realschule” degree after 10 years of formal education . ^c^ equivalent to German general or subject-specific. “Hochschulreife” or “Fachhochschulreife” degree (entrance qualifications for university or university of applied sciences) after eleven. twelve or 13 years of school; * Significant bivariate differences between the mentioned group and non-users at a significance level of p<0.05.; M = Mean; CI = confidence interval; BRS = Brief Resilience Scale; PSS-4 = Perceived Stress Scale; IE-internal = internal control; IE-external = external control; SOP-2 = optimism/pessimism; ASKU = general self-efficacy; weighted to the official statistic; * p<0.05
