# Supplementary material for: Pharmacological neuroenhancement and the ability to recover from stress – a representative cross-sectional survey among the German population
Source: Subst Abuse Treat Prev Policy. 2018 Oct 22;13:37. doi: 10.1186/s13011-018-0174-1 (PMC6198480; doi:10.1186/s13011-018-0174-1)
Supplement: Supplementary file 2 — Correlations between the ability to recover from stress, perception of stress and resilience factors. (DOCX 27 kb) [file 13011_2018_174_MOESM2_ESM.docx]

Additional file 2: Correlations between the ability to recover from stress, perception of stress and resilience factors

|  |  | **BRS** | **PSS-4** | **ASKU** | **IE_ext** | **IE_int** | **SOP-2** |
| --- | --- | --- | --- | --- | --- | --- | --- |
| **BRS** | Coef (N) | 1 (1,128) | -.533** (1,101) | .511** (1,123) | -.449** (1,121) | .450** (1,123) | .505** (1,124) |
| **PSS-4** | Coef (N) | -.533** (1,101) | 1 (1,101) | -.433** (1,096) | .463** (1,094) | -.394** (1,096) | -.512** (1,100) |
| **ASKU** | Coef (N) | .511** (1,123) | -.433** (1,096) | 1 (1,123) | -.419** (1,119) | .620** (1,121) | .458** (1,118) |
| **IE_ext** | Coef (N) | -.449** (1,121) | .463** (1,094) | -.419** (1,119) | 1 (1,121) | -.454** (1,120) | -.437** (1,117) |
| **IE_int** | Coef (N) | .450** (1,123) | -.394** (1,096) | .620** (1,121) | -.454** (1,120) | 1 (1,123) | .447** (1,119) |
| **SOP-2** | Coef (N) | .505** (1,124) | -.512** (1,100) | .458** (1,118) | -.437** (1,117) | .447** (1,119) | 1 (1,124) |

Coef = Pearson correlation coefficient; Predictor variables (BRS, PSS, ASKU, IE, SOP-2)

were z-standardized; BRS = Brief Resilience Scale, PSS-4 = Perceived Stress Scale, ASKU =

Allgemeine Selbstwirksamkeit Kurzskala (General Self-Efficacy Short Scale), IE_ext = External

Control Beliefs, IE_ext = Internal Control Beliefs, SOP-2 = Scale Optimism/Pessimism;

** Correlation´s bivariate significance level of 0.01
